# Data on the experimental characterization of a mobile wick solar still with external condenser tested under the climatic conditions of Rennes, France

**DOI:** 10.1016/j.dib.2024.110395

**Published:** 2024-04-06

**Authors:** Sory Diarra, Sidy Mactar Sokhna, Souleye Faye, Paul Byrne, Ousmane Sow

**Affiliations:** a Laboratoire Eau, Energie, Environnement et Procédés Industriels (LE3PI), Université Cheikh Anta Diop de Dakar, Ecole Supérieure Polytechnique de Dakar, BP, 5085 Dakar-Fann, Sénégal; b Laboratoire Génie Civil et Génie Mécanique, Département Génie Civil Construction Durable, IUT Rennes, 3 rue du Clos Courtel - BP 90422 - 35704 Rennes CEDEX 7, France

**Keywords:** Solar energy, Freshwater, Solar distillation, Energy efficiency, Exergy efficiency

## Abstract

This work evaluates a new prototype of a mobile wick solar still with a passive external condenser designed and manufactured by the company IPFH2O. The systemʼs purpose is to distil non-potable water into potable water using solar energy. It is intended for populations in areas where access to drinking water remains difficult. The tests were conducted over four days between October and December 2021 at the University of Rennes 1 in Brittanyʼs sub-oceanic climate. Manual solar monitoring of the system was conducted at one-hour intervals during the test days. The collected data included temperatures of various components, relative humidity, Global sunshine, and hourly production. This data was then used to calculate the system's energy and exergy yields to evaluate its performance. The collected data is used to determine the system's performance and compare it with other existing solar still. Additionally, it helps to characterize the system's operation and propose technical solutions for optimization.

Specifications Table

**Table:** 

Subject	Energy, Environmental Science
Specific subject area	Energy Engineering and Power Technology, Water Science and Technology
Type of data	Table, Figure, Raw, Analyzed
Data collection	The experiment collected data on the temperature evolution of the solar still's elements, as well as the external ambient environment, relative humidity of the evaporator tank and ambient environment, global sunshine, and hourly production of consumable water. Two types of PT100 thermocouples made by TC-Direct were used to measure temperatures. Surface temperature measurements are taken using PT100 surface thermocouples, while ambient temperature measurements are taken using PT100 ambient thermocouples. Relative humidity is measured using ROTRONIC model CH8303 humidity sensors that operate in the 0 %100 % range. Global solar radiation is measured using an analogue pyranometer. All of these sensors are connected to a RIO-NAPAC analogue data acquisition system, which is controlled by XFLOW software. The acquisition system was used to carry out measurements with a 5-minute step. The final measurement was the amount of consumable water produced by the solar still, which was measured hourly using a graduated container for volume. The data measured have been used to calculate energy and exergy yields.
Data source location	The data was collected at the Rennes 1 University Institute of Technology in the Brittany region of France. The GPS coordinates for the site are Latitude: 48.122359 and Longitude: −1.636877.
Data accessibility	Repository name: Mendeley Data Data identification number: 10.17632/b5zmgghxyj.1 Direct URL to data: https://data.mendeley.com/datasets/b5zmgghxyj/1

## 1 Value of the Data

• The data collected from this new prototype, which combines several technologies such as double glazing, external condenser, and moving mechanism, are valuable for characterizing its operation and performance. Additionally, they can aid in understanding the system's dynamics and the impact of these technological choices.
• Furthermore, the data can contribute to the advancement of the solar still industry. Specialists in the field can use the data to make an informed assessment of the system's performance and compare it with that of well-known systems.
• Solar still researchers can also use the data to develop and validate analytical or numerical models of the solar still, which can help understand how the system works under different climatic conditions.

## 2 Background

Passive solar stills are an affordable and accessible technology, even in remote and impoverished areas. They are proving to be the most practical solution for developing countries that lack drinking water. According to [1], passive solar stills have a reported daily yield and production of 34.4 % and 3.21 kg/m^2^. These data demonstrate the low yield and production of these solar stills, which may explain why they have not been widely marketed to date [2]. However, research projects have been carried out to improve the yield and production of this promising technology. The focus of this data article is mainly on system design and component selection. It is essential to characterize the operation of our prototype solar still, which combines several proven design techniques. Researchers have evaluated the performance of solar stills using experimental methods. Such techniques demonstrate system performance under real-world conditions, and the results are highly regarded by researchers [3]. The daily operation of integrated solar stills is experimentally characterized using comprehensive energy, exergy, economic, and environmental analyses (4E) [4].

## 3 Data Description

### 3.1 Presentation of the solar still with rotating wick and passive condenser

**Fig. 1:**
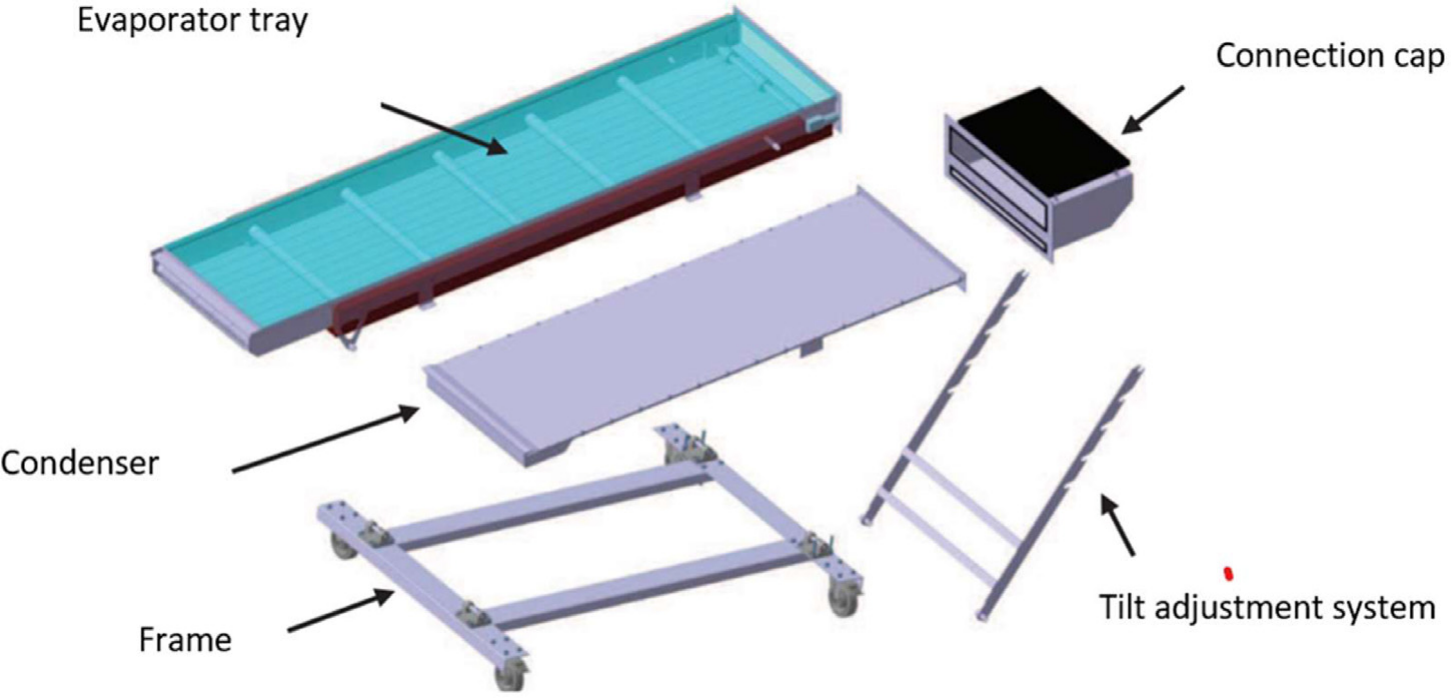
Components of the mobile solar still with passive condenser.

**Fig. 2:**
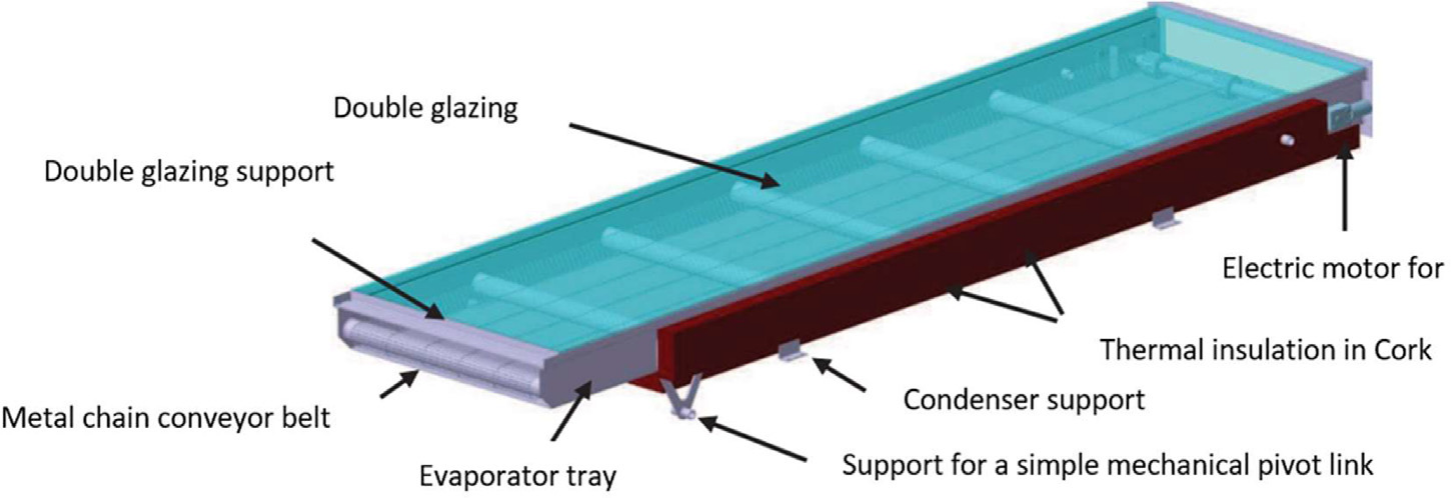
Components of the evaporator tray of the solar still.

**Fig. 3:**
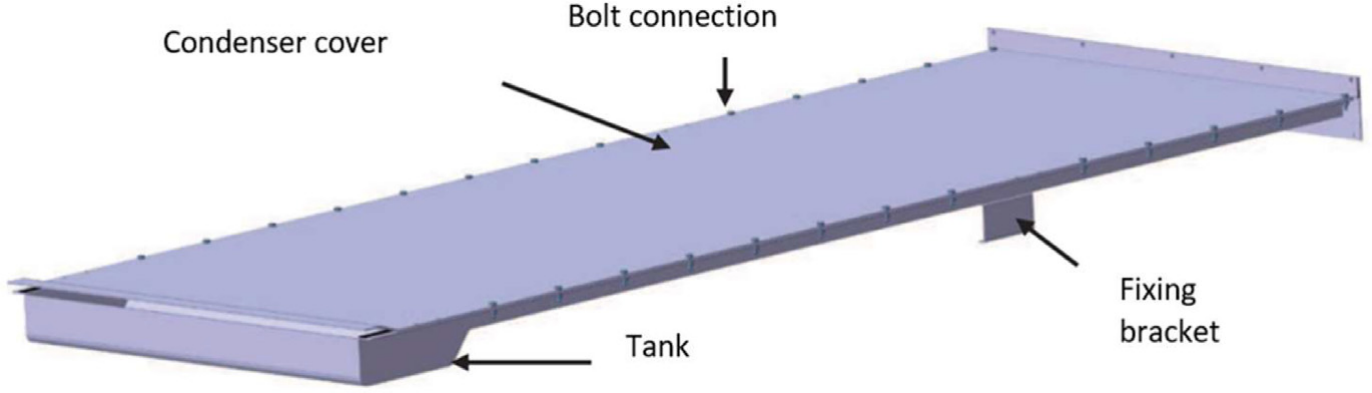
Constituents of the passive condenser of the solar still.

The tested solar still consists of five main parts: an evaporator tray, a condenser tray, a frame, a connection cap, and a tilting system, as shown in Fig. 1. The evaporator tray (Fig. 2) is a box made of 1 mm stainless steel sheet, measuring 2.32 m in length, 0.50 m in width, and 0.076 m in depth. It is thermally insulated with a 2 cm thick polystyrene sheet at the bottom and 1 cm thick on the sides. The polystyrene plates are protected on the outside by another 1 mm thick stainless steel plate. The evaporator tray features a stainless steel metal chain conveyor belt that is powered by a DC motor. This belt drives the wick, which is made of linen fabric in our case. The motor is directly powered by a photovoltaic module to ensure rotation. The metal chain is driven by two shafts equipped with gears located at either end of the bin. Metal cross-members guide the chain. The upper part of the evaporator tray is covered with 5 mm thick clear double glazing and a 1 cm air space. A plastic gasket seals the evaporator tray to the glass. The relatively simple condenser (Fig. 3) consists of a 1 mm thick stainless steel tank measuring 2000 × 570 × 31 mm, covered by a sheet of the same material. The two components are bolted together using 84 screws and nuts.

### 3.2 How it works

**Fig. 4:**
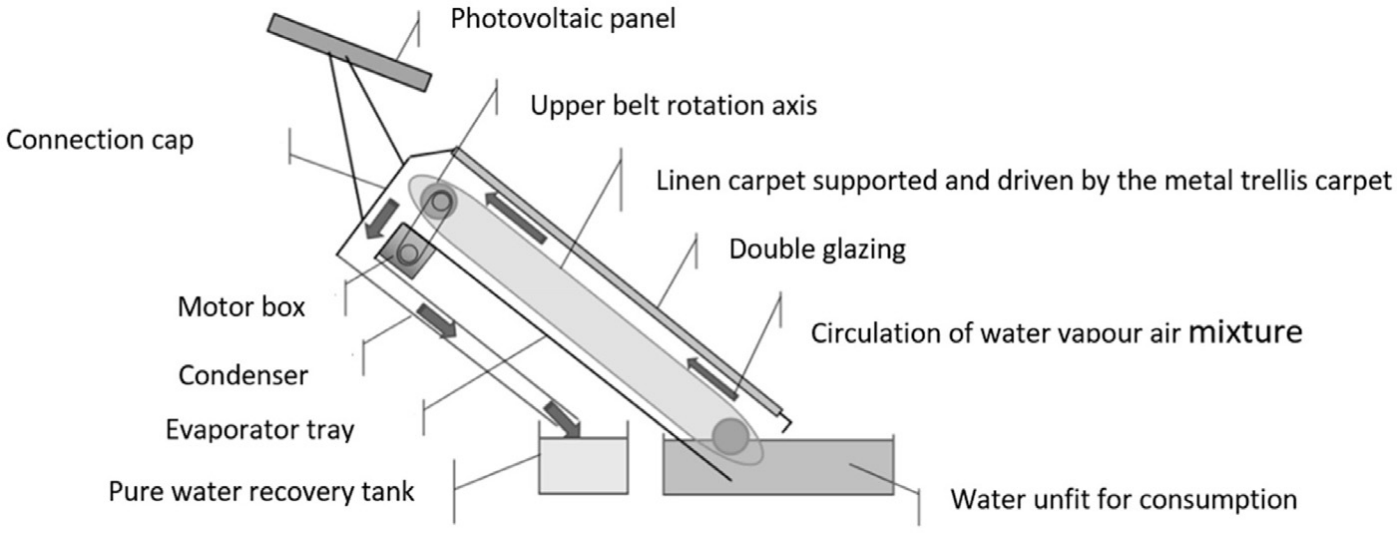
Solar still operating process.

**Fig. 5:**
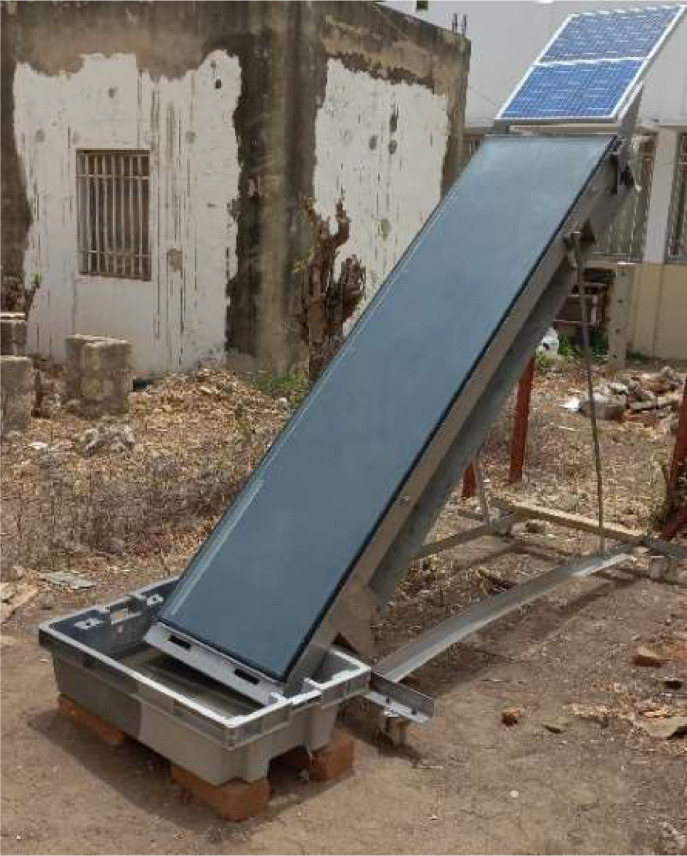
Using the solar distiller.

The wick is represented by a cloth fixed to the metal mesh belt. Rotation of the metal belt causes the fabric to rotate. A tray of dirty or salty water is installed on the lower part of the evaporator tray so that the rotating fabric is in contact with this water when the belt rotates. In this way, the fabric absorbs some of the feed water as it rotates and causes this water to rise into the evaporator tray. At the same time, the part of the fabric in the tray absorbs the incident solar radiation and heats the evaporator tray enclosure by means of the greenhouse effect. Double glazing increases the greenhouse effect in the evaporator and therefore the air temperature in the evaporator enclosure. The warm air allows the water in the fabric to evaporate. This air is then charged with moisture and rises by thermosiphon to the top of the tank and then to the condenser. The air is renewed in the tank through small openings in the lower part of the prototype. Once in the condenser, the moisture-laden water vapour in the air condenses on the walls of the condenser tank. The condensed water flows by gravity to the bottom of the tray, where it is collected through two outlets on either side of the tray Figs. 4 and 5.

### 3.3 Data collected

The following measurements were taken during the tests:

­ Global irradiation on the ground $\left(W/m^{2}\right)$,
­ Ambient air temperature $\left({}^{\circ }C\right)$
­ Ambient air relative humidity (%),
­ Surface temperature of the external glazing $\left({}^{\circ }C\right)$,
­ Surface temperature of the interior glazing $\left({}^{\circ }C\right)$,
­ Surface temperature of the rear base of the evaporator tray $\left({}^{\circ }C\right)$,
­ Temperature inside the evaporator tray $\left({}^{\circ }C\right)$,
­ Relative humidity of the evaporator tray (%),
­ Surface temperature of the condenser tray $\left({}^{\circ }C\right)$,
­ Temperature of the steam leaving the evaporator $\left({}^{\circ }C\right)$,
­ Humidity of the steam leaving the evaporator (%),
­ Temperature of the water entering and leaving the system $\left({}^{\circ }C\right)$,
­ Solar still feed water temperature $\left({}^{\circ }C\right)$,
­ Hourly production of consumable water (ml).

The results obtained from the measurements were used to calculate the energy yields (%) and exergy yields (%) of the solar still.

A total of four trial days have been carried out, on:

­ 14 October 2021
­ 28th October 2021
­ 17th December 2021
­ 21st December 2021

The data from these 4 test days are available on the Mendeley platform at the following URL: https://data.mendeley.com/datasets/b5zmgghxyj/1

**Fig. 6:**
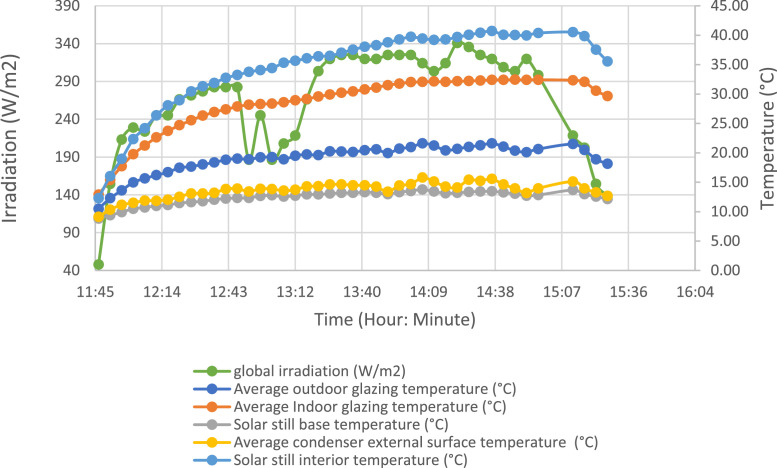
Evolution of global solar irradiation and the temperatures of the system's elements.

The following figure show the evolution of the various system parameters for the test day of 17 December 2021 (Fig. 6).

**Fig. 7:**
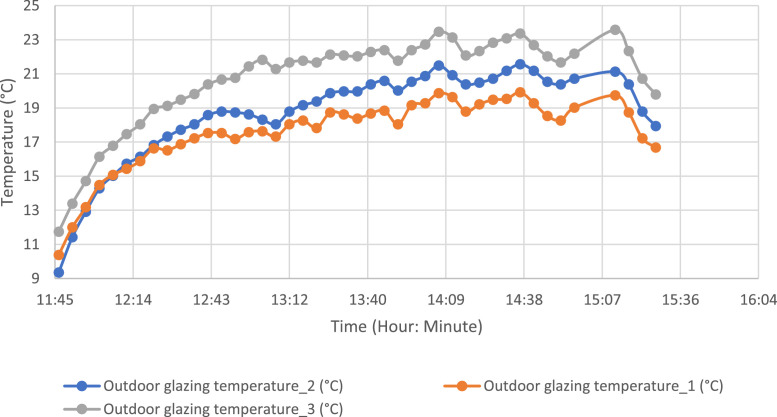
Temperature variation along the outer glazing.

**Fig. 8:**
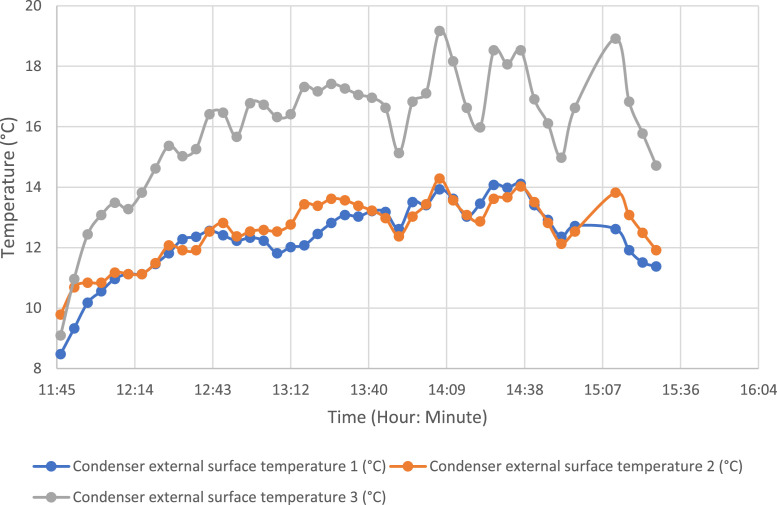
Temperature variation along the external condenser.

To evaluate the temperature gradient along the external glazing, three measurement points were selected. The same method was applied to the external evaporator and condenser tray. Fig. 7, Fig. 8 show the temperature evolution along the glazing and external condenser over time, respectively, with index 1 corresponding to the lowest part.

**Fig. 9:**
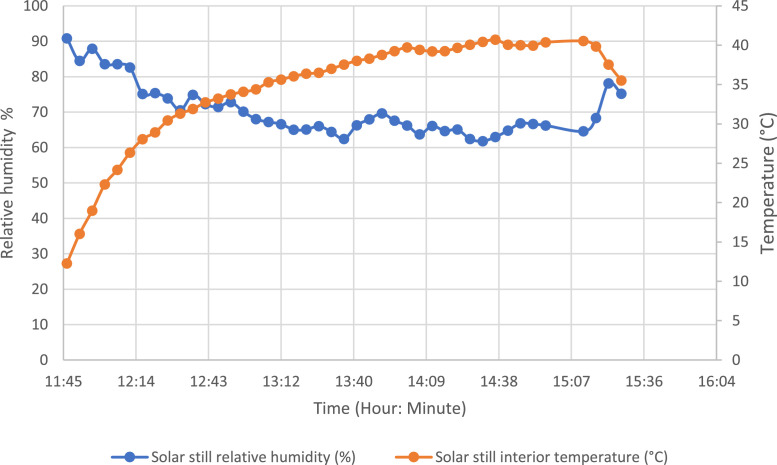
Evolution of temperature and relative humidity of the evaporator tray.

**Fig. 10:**
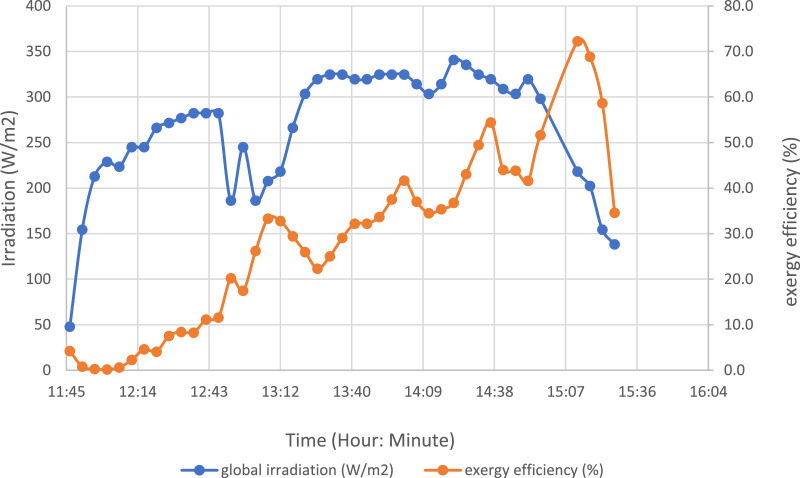
Evolution of global solar irradiation and exergy efficiency.

The energy yields per hour of the solar still were calculated using the results obtained over the 4-day testing period. Additionally, the evolution of the global irradiation and exergy yield for the test day of 17 December 2021 was plotted in Fig. 10. Table 1, Table 2, Table 3, Table 4 show the hourly yields of the solar still calculated for the four test days.

**Table 1:** Evaluation of the hourly energy yield of the solar still for the day of 14/10/2021.

Time (Hour)	Hourly production in kg/h	global irradiation (W/m2)	Feed water temperature (°C)	Wick surface (m2)	Latent heat of vaporization of water (J/kg)	Hourly energy efficiency
13H 14H	0,24	637,02	21,1	1	2,980,407,81	0,31
14H 15H	0,27	630,87	24,87	1	3,177,002,65	0,38
15H 16H	0,315	565,32	25,51	1	3,213,709,75	0,50
16H 17H	0,14	455,84	25,56	1	3,216,618,21	0,27

**Table 2:** Evaluation of the hourly energy yield of the solar still for the day of 28/10/2021.

Time (Hour)	Hourly Fresh water production (kg)	global irradiation (W/m2)	Feed water temperature (°C)	Latent heat of vaporization of water (J/kg)	Wick surface (m2)	Hourly energy efficiency
12h-13h	0,095	512	7,02	2,543,700,49	1	0,13
13h-14h	0,22	566	9,02	2,577,071,16	1	0,28

**Table 3:** Evaluation of the hourly energy yield of the solar still for the day 17/12/2021.

Time (Hour)	global irradiation (W/m2)	Hourly Fresh water production (kg)	Feed water temperature (°C)	Latent heat of vaporization of water (J/kg)	Wick surface (m2)	Hourly energy efficiency
12h-13h	254	0,075	11,4	2,629,165,02	1	0,22
13h-14h	283	0,1	13,12	2,675,182,68	1	0,26
14h-15h	317	0,15	15	2,733,507,55	1	0,36

**Table 4:** Evaluation of the hourly energy yield of the solar still for the day 21/12/2021.

Time (Hour)	Hourly Fresh water production (kg)	global irradiation (W/m2)	Feed water temperature (°C)	Latent heat of vaporization of water (J/kg)	Wick surface (m2)	Hourly energy efficiency
12h-13h	0,095	290	7,02	2,543,700,49	1	0,23
13h-14h	0,22	328	9,02	2,577,071,16	1	0,48
14h-15h	0,26	335	10,07	2,598,395,57	1	0,56

Fig. 9 shows the evolution of relative humidity and evaporator tray temperature in the same graph as a function of time.

## 4 Experimental Design, Materials and Methods

### 4.1 Experiment design and materials

**Fig. 11:**
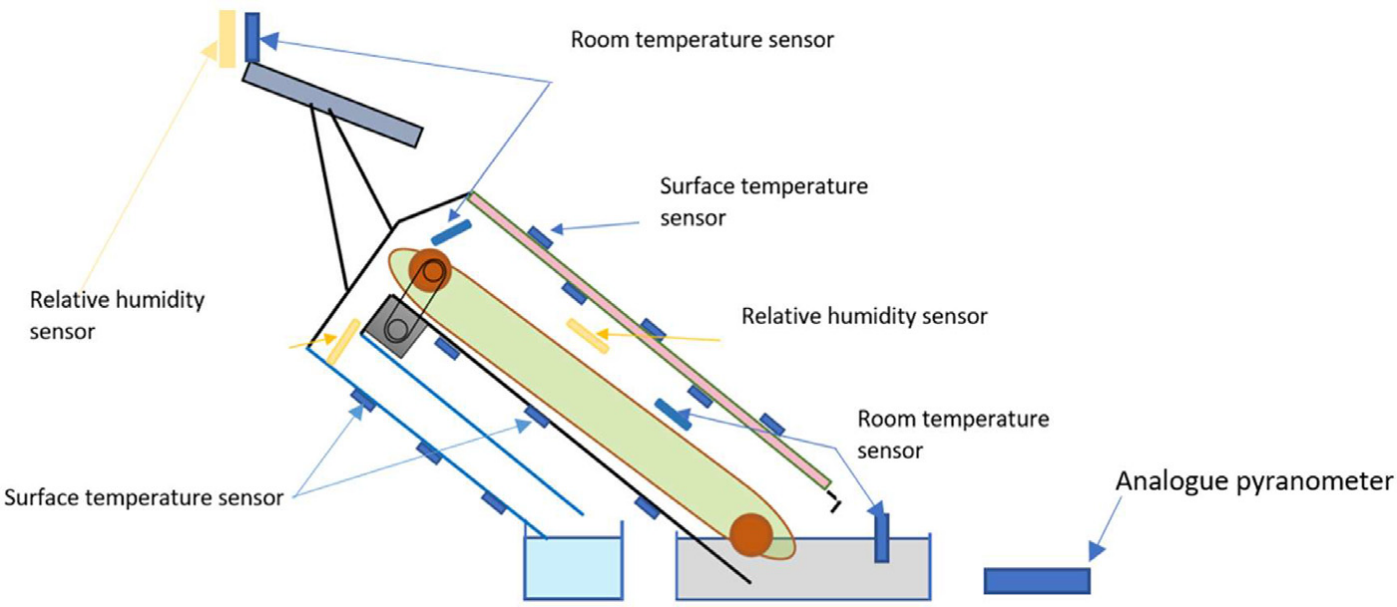
Measurement equipment layout.

**Fig. 12:**
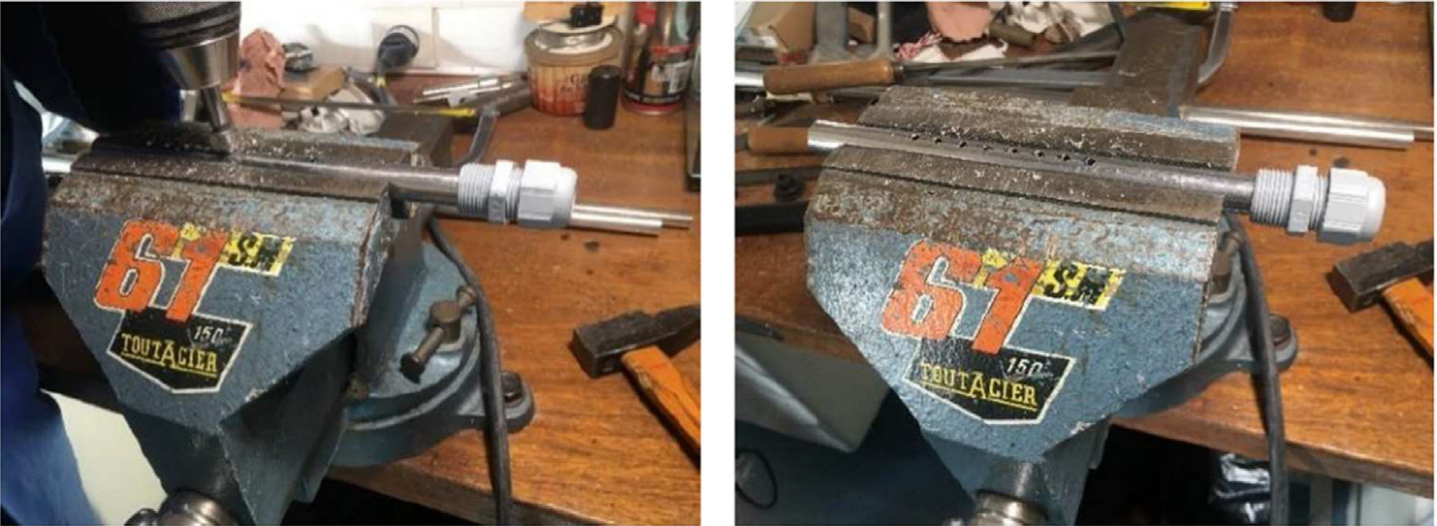
Perforated stainless steel tube for temperature sensor.

To measure the data set defined in the previous section, we carefully select and distribute appropriate instruments. Table 5 below lists the instruments used to measure temperature, relative humidity, and global solar irradiance, along with their respective characteristics. The measuring instruments have been strategically placed on the solar still to obtain the required data. Fig. 11 displays the distribution of the measuring instruments. The surface temperature sensors are used to measure the temperatures of the internal and external glazing, the base of the evaporator tray, and the passive condenser. Ambient temperature sensors are used to measure the temperature inside the evaporator tray, the ambient temperature outside and the feed water to the solar still. The placement of 2 or 3 temperature sensors on the surfaces of the solar still is justified due to the significant temperature gradient that can occur. The surface temperature sensors are protected by aluminum tape to avoid the influence of solar radiation. The temperature sensor inside the tank is protected from solar radiation by a perforated stainless steel tube to allow air circulation, as shown in Fig. 12.

**Table 5:** List and characteristics of measurement equipment.

Instrument : Pyranometer Measurement: Solar irradiation Measurement accuracy: ±5 $W/m^{2}$ Calibration: calibrated with a portable Solari meter	Instrument: Humidity and temperature sensors Manufacturer: Rotronic Model: CH-8303 Measurement accuracy: ±1% humidity and ±0.5 °C for temperature Measuring range: Temperature: −20 °C to 60 °C; humidity: 0 to 100%.
Instrument: Surface temperature sensors Supplier: TC Direct Model: Surface PT100 Measuring accuracy: ±0.5 °C Measuring range: Temperature: −20 °C to 60 °C Location: Surface of glazing, evaporator tray and condenser tray	Instrument: Immersion temperature sensors Supplier: TC Direct Model: PT100 immersion Measuring accuracy: ±0.5 °C Measurement range: Temperature: −20 °C to 60 °C Location: inside evaporator tray, feed water

**Fig. 13:**
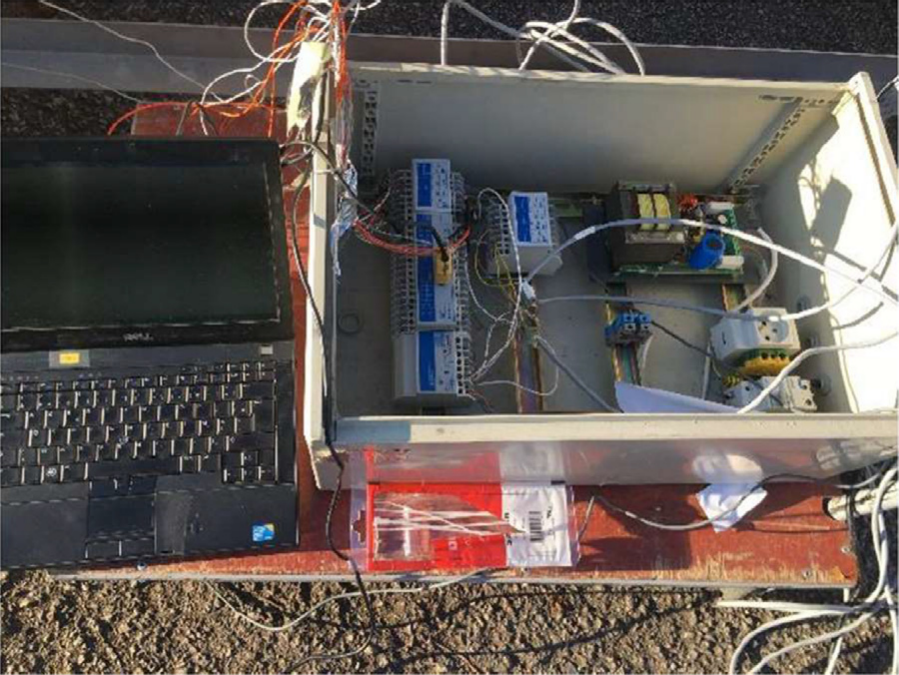
RIO—NAPAC acquisition system.

A data acquisition system is utilized to process and record data. The system comprises a central measuring unit that contains two modules, each of which contains four RIO4AI cards (refer to Fig. 13). Each card has four analogue inputs that can be used to connect sensors. The datalogger is connected to a computer on which an XFLOW Web interface is installed, enabling parameter setting, display, and data retrieval.

Each sensor is connected to a channel on the RIO card, which is recognized as a peripheral in the software. A variable is then configured to allow the measurement to be read. The variable name, device connection, and operation (if required) are provided. XFLOW manages variables using calculation formulas. Whenever a variable is modified or evaluated, its available value corresponds to the most recently calculated value using the corresponding formula. It is also possible to define operations to be performed on variables when they are evaluated, such as slope, mask, differential, pulse, smoothing, and filtering.

When creating a measurement history, the software enables the user to select the minimum recording period. The scale ranges from one second to one month.

The experimental tests followed a protocol consisting of three main stages.

#### 4.1.1 Preparation and adjustment of the prototype

The preparation phase involves removing any elements that may interfere with the measurements, such as dust and impurities on the double glazing and condenser. The electrical cabling between the conveyor belt motor and the solar panels is made using Wago connectors to allow for quick disconnection in case of any issues. Finally, adjust the inclination of the solar panel to match the latitude of the location to ensure maximum solar radiation is received.

#### 4.1.2 Calibration of the sensors and configuration of the acquisition unit

The sensors have been calibrated to minimize measurement errors. The temperature and humidity sensors were calibrated using certified sensors in a controlled environment. The pyranometer, which measures global solar irradiation, was calibrated using an artificial sun. After calibrating the sensors, the acquisition unit was parameterized with the name and characteristics of each sensor, as well as the 5-minute measurement interval.

#### 4.1.3 Launch the test after setting up the sensors and acquisition unit

The gauge sensors have been installed on the system, as illustrated in Fig. 11. To protect the outer surface of each surface temperature sensor from the sun's rays, an aluminum adhesive tape has been used. Similarly, the room temperature sensor is protected from the sun's rays by a perforated stainless steel tube, as shown in Fig. 12. After installing the water supply tank and the purified water recovery tank, orient the solar distiller towards the sun and start the conveyor belt using the Wago motors. Measurements are taken immediately before the conveyor belt is activated. The solar distiller's orientation is adjusted every hour to ensure maximum solar radiation is received continuously. Distilled water production is halted completely when the sun's rays are too weak to rotate the belt, before terminating the acquisition. The obtained data is initially processed and recorded using XFlow software. It is then converted into Excel format for further analysis.

### 4.2 Methods

The tests were conducted during periods of clear sky and moderate ambient temperature. To ensure optimal solar irradiation, the solar still is manually tracked every hour using a wheel attached to the chassis. The tray is inclined at 45° [5].

**Fig. 14:**
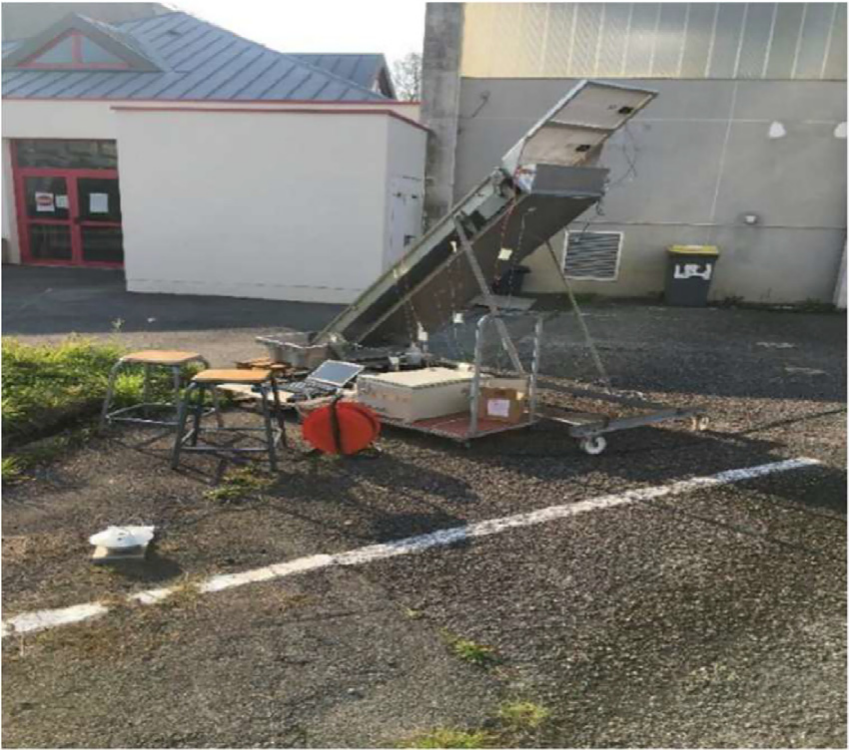
Experimental measurements on the solar distiller with manual sun tracking.

Data is recorded by the acquisition unit in 5-minute intervals, while the quantity of drinking water produced is evaluated hourly (see Fig. 14).

The datalogger measures data which is then processed to calculate the energy and exergy yields. The methodology used to calculate these efficiencies is as follows.

The energy yield per hour of the solar still is calculated using the following formula [6,7]:

(1) $$\eta_{H}=\frac{m_{H}.L}{A_{w.}.I_{t}}$$

(2) $$L=3,\;1615.10^{6}.\lfloor 1-\left(7.616.10^{-4}.T_{w}\right)\rfloor$$

With: $m_{H}\;the\;mass\;of\;water\;produced\;per\;hour\;in\;kg;\;L\;:The\;latent\;heat\;of\;vaporisation\;of\;water$ in (J/kg); $A_{w}$ The surface area of the wick exposed to the sun in $m^{2}$, $T_{w}$ the temperature of the wick in °C and $I_{t}$ global incident irradiation in W/$m^{2}$.

The exergy efficiency of solar stills is defined as follows [8]: et

(3) $$\eta_{exer}=\frac{Ex_{out}}{Ex_{inp}}\;$$

$Ex_{out}\;\left(Exergy\;output\right)$ corresponds to the evaporation exergy between the wick and the interior glazing $Ex_{e,\;w-gi}$. $Ex_{inp}$ (Input Exergy) corresponds to the exergy of solar radiation absorbed by the wick $Ex_{sun}$ [9].

(4) $$Ex_{e,\;w-gi}=h_{e,w-gi}.\;A_{w.}.\left(T_{w}-T_{gi}\right).\left(1-\frac{T_{a}}{T_{w}}\right)\;\left[8\right]$$

With:

$h_{e,w-gi}$ the evaporative heat transfer coefficient in (W/m2.K); $T_{w}$ the temperature of the wick in °C; $T_{gi}$ the temperature of the interior glazing in °C and $T_{a}$ ambient temperature in °C.

(5) $$h_{e,w-gi}=0,\;016,\;273\;h_{c,w-gi}.\left(\frac{P_{w}-P_{gi}}{T_{w}-T_{gi}}\right)\;\left[10\right]$$

With:

$h_{c,w-gi}$ the convective heat transfer coefficient between the wick and the interior glazing in (W/m2.K); $P_{w}\;and\;P_{gi}$ respectively the partial pressure at the surface of the wick and that of the inner pane.

(6) $$P_{w}=\;exp\left(25,\;317-\frac{5144}{T_{w}+273}\right)\;\left[8\right]$$

(7) $$P_{gi}=\;exp\left(25,\;317-\frac{5144}{T_{gi}+273}\right)\;\left[8\right]$$

(8) $$h_{c,w-gi}=0,\;884\left[\left(T_{w}-T_{,gi}\right)+\frac{\left(P{}_{w}-P{}_{gi}\right)\left(T_{W}+273,\;15\right)}{268900-P_{w}}\right]\;\left[8\right]$$

(9) $$Ex_{sun}=A_{w.}\;I_{t}.\left(1-\frac{4}{3}\right).\left(\frac{T_{a}+273}{T_{S}}\right)+\frac{1}{3}.\left(\frac{T_{a}+273}{T_{S}}\right)\;.\;\text{4}\;\left[8\right]$$

With $T_{S}$ the temperature of the sun which is approximately equal to 6000 K.

$T_{w}$ is taken to be equal to the temperature of the evaporator mixture – 2 °C [10], [11]

### Limitations

During data collection, we encountered limitations due to difficult climatic conditions.

The tests were carried out in Brittany, an area with infrequent sunny weather, especially during the period of the tests. As a result, our measurements started late in the day, with some tests beginning at around 10–11am. The data collected sometimes shows that the system is completely shut down during cloudy spells.

Additionally, the solar distiller has difficulty starting distillation on days with low outside temperatures, even if the solar radiation measured is correct.

The solar distiller is mounted on wheels to facilitate manual solar tracking. However, moving the entire unit, including the solar distiller, feed water tank, and data acquisition system, is a complicated process that requires the assistance of three people.

### Ethics Statement

This data collection does not involve humans, animals, and is not sourced from social media networks.

## CRediT authorship contribution statement

**Sory Diarra:** Conceptualization, Methodology, Investigation, Data curation, Software. **Sidy Mactar Sokhna:** Visualization, Investigation, Validation. **Souleye Faye:** Visualization, Validation. **Paul Byrne:** Investigation, Software. **Ousmane Sow:** Visualization, Validation.

## Data Availability

Data on the experimental characterization of a mobile wick solar still with external condenser tested under the climatic conditions of Rennes, France (Original data) (Mendeley Data)
